# Polyphenism of visual and chemical secondary sexually-selected wing traits in the butterfly *Bicyclus anynana*: How different is the intermediate phenotype?

**DOI:** 10.1371/journal.pone.0225003

**Published:** 2019-11-18

**Authors:** Doriane Muller, Benjamin Elias, Laurent Collard, Christophe Pels, Marie-Jeanne Holveck, Caroline M. Nieberding

**Affiliations:** 1 Group of Evolutionary Ecology and Genetics, Biodiversity Research Centre, Earth and Life Institute, Université Catholique de Louvain (UCLouvain), Louvain-la-Neuve, Belgium; 2 Institute of Condensed Matter and Nanosciences, Université catholique de Louvain (UCLouvain), Louvain-la-Neuve, Belgium; Texas State University, UNITED STATES

## Abstract

Polyphenism is a type of phenotypic plasticity supposedly adaptive to drastic and recurrent changes in the environment such as seasonal alternation in temperate and tropical regions. The butterfly *Bicyclus anynana* shows polyphenism with well-described wet and dry seasonal forms in sub-Saharan Africa, displaying striking morphological, physiological and behavioural differences in response to higher or lower developmental temperatures. During the seasonal transition in the wild, the intermediate phenotype co-occurs with wet and dry phenotypes. In this study, we aimed to characterize the secondary sexually-selected wing traits of the intermediate form to infer its potential fitness compared to wet and dry phenotypes. Among the previously described wing morphological traits, we first showed that the area of the fifth eyespot on the ventral hindwing is the most discriminant trait to identify wet, dry and intermediate phenotypes in both sexes. Second, we characterized the intermediate form for two secondary sexually-selected wing traits: the area and UV reflectance of the dorsal forewing pupil and the composition of the male sex pheromone. We showed that values of these two traits are often between those of the wet and dry phenotypes. Third, we observed increasing male sex pheromone production in ageing dry and wet phenotypes. Our results contrast with previous reports of values for sexually-selected traits in wet and dry seasonal forms, which might be explained by differences in rearing conditions or sample size effects among studies. Wet, dry and intermediate phenotypes display redundant sexually dimorphic traits, including sexually-selected traits that can inform about their developmental temperature in sexual interactions.

## Introduction

Phenotypic plasticity, which occurs when a genotype produces different phenotypes in response to different environments, has been acknowledged as a potentially important mechanism of rapid adaptation to predictably varying environments [1–3]. Polyphenism is a case of phenotypic plasticity where discrete, qualitatively differentiated, phenotypes are found in contrasting environments [4]. Polyphenism has been mostly described on the basis of morphological traits; for example aphids show dispersal polyphenism with winged and wingless forms respectively reproducing sexually and asexually, depending on crowding, seasonality, host plant and interspecific interactions (reviewed in [5,6]). Similarly, some moths and butterflies display strikingly different seasonal wing patterns in response to seasonal alternation [7]. Polyphenism in sexual traits is documented in many species. For example, horn size is a polyphenic secondary sexual trait linked to larval diet in some beetles; sex determination is environmentally-induced by temperature variation in reptiles and fishes (reviewed in [5,6,8–10]). Polyphenism can also involve physiological and behavioural phenotypic differentiation, such as alternations of gregarious and solitary generations in locusts in response to biotic cues (e.g. local crowding; [10]). Morphological, physiological and behavioural polyphenism is found across multiple taxonomic groups [11,12]. It is usually considered to be adaptive by allowing phenotypic matching to contrasted environments that alternate recurrently in time and can be predicted from reliable environmental cues [5,6,13–16].

**Fig 1:**
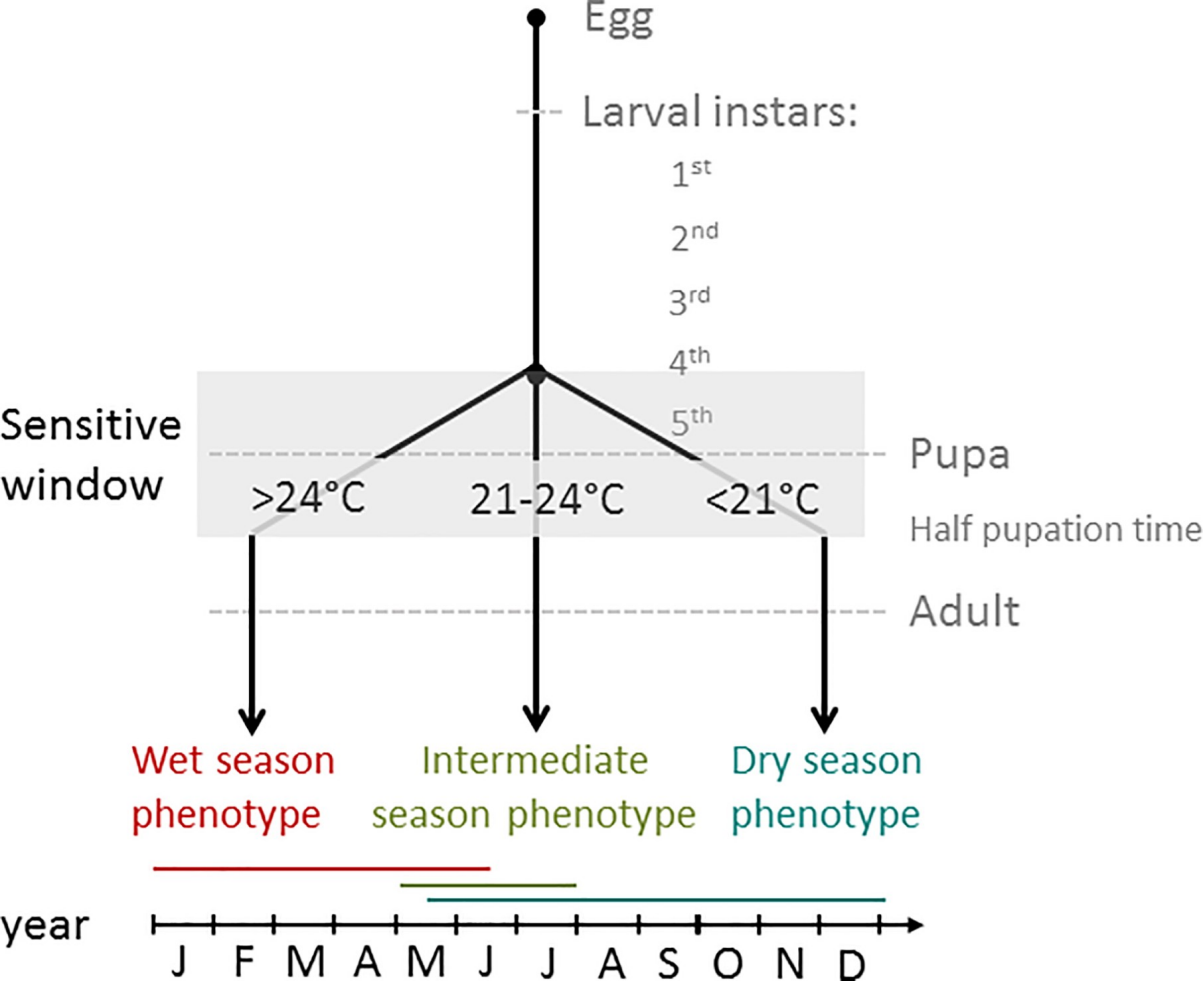
Temperature ranges leading to the production of wet, intermediate and dry season forms and their occurrence across the year in Malawi [7,19]. Seasonal forms occurrence and changes throughout season may vary according to specific climatic conditions in other parts of the species distribution area [24]. Red, green and blue lines show when WS, IS and DS forms are found in the field in Malawi, respectively. There is no data about the IS presence in the field at other times of the year.

**Fig 2:**
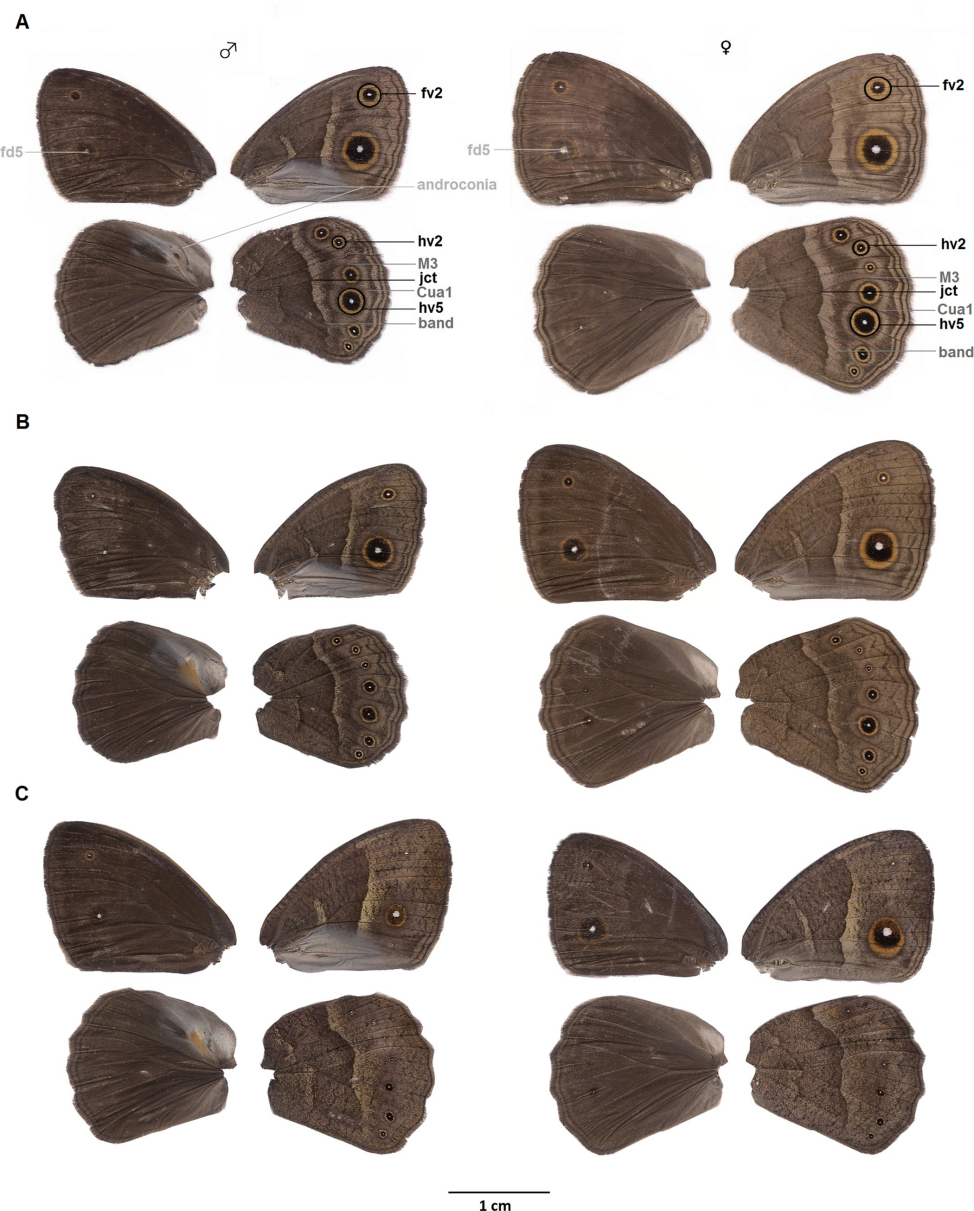
**The dorsal (first and third columns) and ventral (second and fourth columns) parts of female (right) and male (left) wings of the (A) wet, (B) intermediate and (C) dry seasonal forms.** The wing morphological traits most frequently measured to quantify polyphenism in published literature are highlighted in black: areas of (fv2) the anterior eyespot on ventral forewing, (hv2) the second eyespot on ventral hindwing and (hv5) the fifth eyespot on ventral hindwing, and (jct) the junction of the two veins (M3) and (Cu1a) to (band) distal band highlighted in grey. The following secondary sexual traits are written in light grey: (fd5) the relative brightness and area of the anterior eyespot on dorsal forewing pupil and (androconia) the organs producing the 3 MSP components.

The butterfly *Bicyclus anynana* (Butler 1879; Nymphalidae) has become a model for the study of seasonal polyphenism [17] and displays wet (“wet seasonal”, “WS” hereafter) and dry (“dry seasonal”, “DS” hereafter) seasonal forms depending on high, or low, developmental temperature, respectively. These two seasonal forms match the temporal alternation of seasons in sub-tropical Africa [7,18] with two generations of WS phenotype successively covering the entire warmer wet season and one DS generation surviving the whole colder dry season (Fig 1)[7,19]. They display striking morphological differences in wing patterns (Fig 2), as well as physiological and behavioural differences [19–21]. Morphologically, the WS form has numerous eyespots on the ventral sides of its wings, which deflect predators’ attacks and confer an adaptive advantage in the luxuriant vegetation of the wet season [15,22]. The DS form displays a more cryptic phenotype with smaller eyespots and a more uniform brown colour that conceals individuals in the environment typical of the dry season [7]. Physiologically, the adult DS phenotype has a higher resistance to starvation than the WS form, thanks to its lower resting metabolic rate and higher fat body accumulation, likely to adapt to the low food availability in nature during the dry season [7,19,21]. DS females postpone reproduction during dry season probably because the wet season is more adequate for offspring survival [7]. Successful breeding in the wet season for DS females could be promoted by mating with DS males, which improves their survival and fecundity prospects [23]. Behaviourally, WS males show more active courtship and general activity than DS males, which coincides with the observation that most matings occur during the wet, reproductive, season in the wild [7,20,23].

Seasonal polyphenism in *B*. *anynana* appears to contrast with most cases of documented polyphenism where intermediate phenotypes are usually rare and thus considered as maladaptive [25,26]. The intermediate phenotype in *B*. *anynana* (“intermediate seasonal”; “IS” hereafter) coexist in nature with the WS and DS forms during the seasonal transition, and can become for weeks the most abundant form as they may represent up to 75% of all individuals [7,27]. So far, the fitness associated to the IS phenotype in *B*. *anynana*, including fitness benefits associated to mating with IS males, remains unknown. Yet, we expect that the lower year-round abundance (8.3%) of the IS phenotype compared to more extreme phenotypes (DS: 38.1%; WS: 53.6%) indicates that the IS phenotype is maladapted compared to the WS and DS phenotypes most of the year [27]. We also expect that the IS phenotype displays phenotypic traits that are distinctive from WS and DS forms, providing information on their intermediate developmental thermal history [28,29] and providing room for negative selection in the wild. Hence, *B*. *anynana* may provide a relevant model system for assessing the roles of natural and sexual selective pressures on the elimination or the maintenance of intermediate phenotypes in the evolution of polyphenism.

Here, we aimed at characterizing several phenotypic traits of WS, DS and especially of IS forms to assess how the IS phenotype differs from WS and DS phenotypes. This is a first step to evaluate the potential (mal) adaptive value in the wild of the IS phenotype. Our specific objectives for this were two-fold: (i) identify the seasonal wing morphological trait with the most discriminative power in *B*. *anynana* to easily differentiate IS from WS and DS forms in both sexes; (ii) quantify the IS phenotype with a suite of phenotypic traits that either evolve under natural or sexual selection. In this paper, we also focused on the relation between environmental (developmental temperature) and intrinsic (age) factors on the expression of a chemical sexually selected trait, the male sex pheromone (MSP hereafter), in these butterflies living for at least three months in the field [19]. For this, our third specific objective was to: (iii) quantify age-related changes in the MSP composition between DS and WS seasonal forms. For the first objective, previous studies of *B*. *anynana* used various methods to discriminate between forms, from visual qualitative classifications in the field to morphological and colour quantitative measurements of wing traits (reviewed in S1 Table). We selected a number of wing morphological traits according to both their high frequency of use and good discriminative power between WS and DS forms in previous studies (S1 Table). We hypothesized that *B*. *anynana* phenotypes are best discriminated by ventral eyespot areas, especially the fifth eyespot which is often used in studies quantifying *B*. *anynana* seasonal phenotypes (S1 Table). Indeed, ventral eyespots are known to be strongly influenced by developmental temperature [30]. For the second objective, we characterized the IS form for some additional, secondary sexually-selected, traits. In *B*. *anynana* WS and DS forms, these traits (reviewed in [31] are: (i) the area and ultra violet (“UV” hereafter) reflectance of the white pupils at the centre of the eyespots located on the dorsal forewing [32,33]; (ii) the composition of the MSP formed by three components [(Z)-9-tetradecenol, hereafter “male sex pheromone 1”, or MSP1), hexadecanal (hereafter MSP2) and 6,10,14-trimethylpentadecan-2-ol (hereafter MSP3)] [34–36]; and (iii) courtship activity [20,37–39]. In WS males, sexual selection stabilizes the area of dorsal forewing eyespot around the mean population value [32], while there is directional sexual selection towards increasing values of MSP components, UV reflectance of pupils and courtship activity [20,32,34–40]. Additional wing morphological traits may be under stabilizing sexual selection, such as the number of eyespots on dorsal hindwings [41]. Sexual selection has mostly been studied in the WS form [32,34,35,42], which supposedly performs most reproduction events in the wild [7]. Yet, these three secondary sexual traits were also shown to be plastic between the DS and WS forms [20,23,43] and to correlate to a reversal in the sex roles between seasonal forms with DS males becoming the choosy sex and DS females competing for male access [20,23]. Here we quantified variation in MSP composition and in the area and UV reflectance of dorsal forewing eyespots in both sexes and across the range of developmental temperatures producing the WS, DS and IS forms. The characterization of the secondary sexually-selected traits in the IS form should help evaluating its potential fitness among a heterogeneous population of butterflies with various phenotypes as it occurs during seasonal transition in nature. We expected that IS individuals should display intermediate values of sexually-selected traits compared to WS and DS individuals and that variation in these sexually-selected traits across seasonal forms should allow individuals to identify the developmental history of potential mates, when the seasonal forms co-occur in natural environment. This is likely under sexual selection as mating with DS males conveys higher fitness benefits to females than mating with WS males [23]. The fitness effects of mating with IS males remain unknown, but may be low given that IS individuals are not found during the dry nor the wet season in the field [26]. Regarding our third objective, several studies analysed age-related changes in MSP composition in the WS phenotype [35,44], but only Nieberding *et al*. [45] quantified MSP components in both WS and DS phenotypes at different ages, without focusing on this aspect. Hence, we re-analysed data from published literature [45]. Here we assessed potential differences in developmental reaction norms of MSP production with increasing age for these two seasonal forms. We expected MSP composition to significantly change with age in both DS and WS males, as previously found in WS males [35,44].

## Materials and methods

### Production of the adult seasonal forms

The outbred laboratory stock population of *Bicyclus anynana* at the University of Louvain-la-Neuve was established from 80 gravid females collected in Malawi (Nkhata bay) in 1988 [17]. At each generation, 400 to 600 adults breed to produce the next generation to maintain a high level of heterozygosity [46]. Except if stated otherwise, our stock and experimental populations were reared in a large climate-controlled room (5.8 x 5.7m and 2.27m high) at 27°C (±0.84°C; SD), 70% (±3.01%; SD) of relative humidity, and 12:12 photoperiod representing the average natural environment experienced during the wet season in Malawi [17].

In two experiments, we reared larvae in groups of 40 to 50 individuals and we kept humidity and photoperiod similar to the standard rearing environment (27°C) in all thermal treatments. In the first experiment (experiment A), we reared larvae either in the standard rearing environment (27°C) or in incubators (SANYO MIR554, SANYO MLR351H) at 17°C (±0.79°C; SD), 21°C (±0.14°C; SD), and 23°C (±0.21°C; SD). The temperatures of 17°C and 27°C produce typical phenotypes of the dry and wet season, respectively [7,23], while temperatures above 20°C and below 24°C produce the IS form [19,21]. Eggs of all thermal treatments were first reared at 27°C. Then, larvae were placed in the different thermal treatments from 15 days after egg collection until the second half of their pupation stage. Indeed, the temperature experienced during this developmental window (i.e. from the fourth larval instar) determines the expression of the adult phenotype [7,18,20,21]. We reduced developmental duration by transferring pupae back at 27°C either 5 days after pupation for larvae reared at 21°C (IS-21/27) and 23°C (IS-23/27), or 10 days after pupation for larvae reared at 17°C (DS-17/27). These durations represent the expected half-pupation time for each seasonal form, after which the sensitive window for determining the seasonal form based on temperature is closed [20,21]. In the second experiment (experiment B), we re-used already published data from Nieberding *et al*. [45] to quantify lifetime changes in MSP production across WS and DS forms. Here the larvae were reared either in the standard rearing environment (27°C) or at 20°C in an incubator (SANYO MLR351H), and the pupae and adults after emergence were kept at the same temperature as the one experienced during development (27°C: WS-27/27; 20°C: DS-20/20).

In both experiments, each day, newly emerged adults were sexed, individually marked on their ventral forewing with an indelible felt-tip pen. Same-sex virgin groups were kept in cylindrical cages (diameter of 30 cm, height of 38 cm) at similar densities (6.57 ± 3.63 females per cage and 6.05 ± 3.10 males per cage; mean ± SE). Throughout, we fed larvae and adults *ad libitum* with 3-week old leaves of maize *Zea mays* (24°C, 60% relative humidity) and slices of fresh and moist, organic banana *Musa acuminata* replenished every two days, respectively. Virgin adults were killed at 8 days of age in experiment A and at either 3, 7, 14, 21 or 28 days of age in experiment B, by immediately freezing individuals in entomological envelopes at -80°C.

### Quantification of the wing polyphenic traits

Butterfly wings were carefully cut on ice using dissecting scissors rinsed with isohexane. Morphological traits were quantified using the right fore- and hindwings and MSP composition was assayed using the left fore- and hindwings.

Digital pictures (768x576 pixels) of the ventral side of the wings from experiment A were obtained under artificial light source on a white background with a ruler (1 mm), using the software UniversalCam 3.5 linked to a binocular microscope (LEICA) fitted with a Sony camera (SSC-DC198p) [24]. We quantified the area of the four, most often used, wing traits in the literature to measure wing polyphenism in *B*. *anynana* (S1 Table): (1) the anterior eyespot on the ventral forewing (fv2), (2) the second eyespot on the ventral hindwing (hv2), (3) the fifth eyespot on the ventral hindwing (hv5) and (4) the distal band (jct) (Fig 2A). In ImageJ software (1.4.3.67) [47], the areas of the fv2, hv2, and hv5 eyespots were obtained with the Polygon selection. The size of the distal band was measured as the distance from the junction of the two veins Cu1a and M3 to distal band [48,49] (Fig 2). We measured wing size by calculating the Euclidian distance between the centroids (obtained from the Wand tracing tool; mode: 8-connected, tolerance~40) of the fv2 and posterior eyespots on ventral forewing, and of the hv2 and hv5 eyespots on ventral hindwing [50]. The sizes of all eyespots and of the distal vein were corrected by wing size to reduce inter-individual variation due to overall variation in body size. The proxy of wing size was squared when used to correct eyespot area to standardize these ratios.

Next, we photographed the dorsal sides of wings from experiment A under a uniform Ultra Violet light having wavelengths in the range of 356-374nm (custom flash light 365nm Nichia Power UV LED with a holographic condenser system). We measured two visual secondary sexual traits: the UV reflectance (as relative brightness) and area of the white pupil of the posterior fd5 eyespot on forewing (Fig 2). Pictures were taken with a modified UV-sensitive camera (Nikon D200, 10 Mpix; settings: Mode Manual, Aperture 11; f/3.5; 1/60s; ISO-400; white balance: 2500k; exposition: +1.7, [51,52]), equipped with an UV transmitting internal filter extending the spectrum down to 300nm and with an UV transmitting macro lens (Nikon Nikkor Ai 35 mm f/2.8; >320nm) fitted with a Baader U-filter 2 selecting UV between 320 and 380nm (350nm center, ca. 80% peak transmission). A custom black-grey-white stepped reflection standard (4 Spectralon blocs with 10/25/50/95% of flat reflectance from UV to IR) allowed standardizing light intensity variation across pictures. Following Papke et al [53], RGB pictures (jpg, 3872x2592 pixels) were converted to grayscale (HSB stacks). We calculated the mean brightness of the UV-reflective pupil area from the brightness stack using the wand tool (mode: 8-connected, tolerance: 25) to trace the area and the histogram function to obtain mean luminosity (on a scale of 0–255). We corrected minor variations in UV lighting conditions between pictures by calculating the standardized brightness of each picture based on the deviance of brightness mean (calculated from the full sample) as follows: “brightness of each fd5 pupil of the focal picture” minus the deviation, which was the absolute value of |“brightness mean of the reference of all pictures” minus “brightness of the reference of the focal picture”| [54]. The reference was an area of 16040 pixels taken at the same position on all pictures on the 50% flat reflectance Spectralon bloc.

We quantified the amount of the three MSP components in experiment A and B following Nieberding *et al*. [34]. In experiment A, fore- and hindwings were placed for 10 minutes in 350μL of isohexane (97% for HPLC, VWR) for extraction with an internal standard (palmityl acetate at 10ng/μL from Sigma Aldrich). After extraction, samples were filtered with glass Pasteur pipettes containing a piece of glass wool and analysed using a gas chromatograph (Agilent GC7890A) with a flame ionisation detector (Agilent Technologies, Belgium; GC-FID) with injection of 1 μL in a splitless mode with an ALS autosampler (Agilent autosampler 7693). Once temperature at injector held 250°C and a pressure of 14.23psi, the solution was conducted in a DB5 Column (30m x 0.32mm x 0.25μm) with a hydrogen flow of 2mL/min as carrier gas. The oven temperature firstly reaches 75°C for 3 min, followed by an increasing temperature of 20°C/min to 220°C, and then by a final ramp of 30°C/min held to a temperature of 300°C for 7 min. Detector was held at 250°C with a flame composition of 30mL/min of hydrogen, 350mL/min of air and of 20mL/min of a nitrogen makeup gas. Hydrogen was produced by a generator (PEAK Scientific PH300) with ultrapure water generated by Thermo Scientific Barnstead Easypure II. For experiment B, differences from above include that wings were soaked for 5 min in a solution of 600μL with 1ng/μL of palmitic acid used as internal standard in hexane and samples were run in a Hewlett-Packard 6890 series II GC-FID with nitrogen as carrier gas through a HP-1 column (for more details refer to [45]). We checked the exact retention time of the three MSP components in running a mixture of the three synthetic components each day before running samples.

### Statistical analyses

All statistical analyses were carried out using R Studio (3.4.2) and graphs plotted with the R package ggplot2 [55,56].

#### 1. Variation in a selection of wing morphological traits, including sexually-selected traits, across seasonal forms

In the first analysis step, we assessed the level of polyphenism of a selection of wing morphological traits (hv5, fv2, and hv2 areas, and jct) and two visual secondary sexual traits (the area of the dorsal forewing white pupil (fd5 eyespot) divided by wing size and fd5 relative brightness) from experiment A. Here we aimed at finding the trait with the most discriminative power to distinguish WS, IS and DS phenotypes, which we defined as “the most polyphenic trait”. The MSP composition was not included in this analysis as it is a male-specific trait. First, we evaluated the correlation between pairs of these traits using a Spearman’s rank correlation. Second, we identified the most variable trait(s) in response to developmental temperatures using a linear discriminant analysis (LDA) (R package FactoMinR) on 175 individuals (DS-17/27 = 45, IS-21/27 = 41, IS-23/27 = 44, WS-27/27 = 45).

In the second step, we assessed variation of wing morphological traits across developmental temperatures, sex and their interaction using orthogonal polynomial regressions by including a quadratic term for developmental temperature [R function poly(x,2)] and checked whether the trait considered as having the most discriminative power, had the reaction norm with the highest slope. Sample sizes per sex and developmental temperature ranged from 24 to 33 individuals (Table 1).

**Table 1:** Descriptive statistics of the wing traits per developmental temperature (17°C, 21°C, 23°C, 27°C) in experiment A.

			DS-17/27		IS-21/27		IS-23/27		WS-27/27	
Sex	Trait type	Wing traits	mean±1SD	N	mean±1SD	N	mean±1SD	N	mean±1SD	N
Males	Morphological	hv5 area (mm^2^)/wing size (mm^2^)	0.03±8.66e-3	27	0.05±0.02	27	0.09±0.02	33	0.11±0.02	28
		hv2 area (mm^2^)/wing size (mm^2^)	4.28e-3±1.90e-3	27	9.33e-3±3.69e-3	27	0.02±5.70e-3	33	0.02±4.86e-3	28
		fv2 area (mm^2^)/wing size (mm^2^)	0.02±5.98e-3	27	0.04±0.01	27	0.06±0.02	33	0.09±0.02	28
		jct (mm) /wing size (mm)	0.07±0.02	27	0.07±0.02	27	0.07±0.02	33	0.05±0.02	28
Females	Morphological	hv5 area (mm^2^)/wing size (mm^2^)	0.02±6.82e-3	30	0.05±0.02	26	0.07±0.02	25	0.11±0.02	24
		hv2 area (mm^2^)/wing size (mm^2^)	2.33e-3±1.14e-3	30	5.82e-3±2.33e-3	26	0.01±4.71e-3	25	0.02±4.83e-3	24
		fv2 area (mm^2^)/wing size (mm^2^)	0.01±5.10e-3	30	0.03±0.01	26	0.05±0.02	25	0.07±0.02	24
		jct (mm) /wing size (mm)	0.08±0.02	30	0.08±0.03	26	0.06±0.02	25	0.05±0.03	24
Males	Sexually-selected traits	fd5 area (mm^2^) /wing size (mm^2^)	2.00e-3±1.26e-3	29	2.25e-3±1.15e-3	29	2.86e-3±1.60e-3	24	3.12e-3±1.36e-3	31
		fd5 relative brightness	139.51±25.65	31	139.77±24.47	30	114.36±38.58	30	112.55±29.74	35
		MSP1 (ng/individual)	2794.66±1051.70	31	3397.95±1237.19	33	4024.96±1598.33	31	4337.01±1675.47	37
		MSP2 (ng/individual)	1216.26±495.74	31	1124.86±447.73	33	1190.62±335.94	31	1204.18±409.20	37
		MSP3 (ng/individual)	10988.28±4313.78	31	13008.98±4338.98	33	16933.32±6568.31	31	17657.83±6959.18	37
		MSP2/MSP1	0.51±0.22	30	0.38±0.22	33	0.34±0.21	30	0.31±0.15	37
		MSP2/MSP3	0.13±0.06	30	0.1±0.05	33	0.08±0.06	30	0.08±0.05	37
		MSP1/MSP3	0.26±0.05	31	0.26±0.06	33	0.25±0.08	31	0.25±0.05	37
Females	Sexually-selected traits	fd5 area (mm^2^) /wing size (mm^2^)	4.97e-3±1.44e-3	32	5.80e-3±1.92e-3	29	5.35e-3±1.69e-3	29	6.74e-3±1.77e-3	29
		fd5 relative brightness	143.71±24.86	32	157.44±24.93	33	155.00±21.51	33	152.36±23.20	33

DS-17/27, IS-21/27, IS-23/27, and WS-27/27 respectively mean Dry Season phenotype produced with a developmental temperature of 17°C, Intermediate Season phenotype obtained with a developmental temperature 21°C or 23°C, and Wet Season phenotype with a developmental temperature of 27°C, all kept at 27°C at the adult stage. SD: Standard Deviation; N: sample size; MSP: Male Sex Pheromone; hv5: fifth eyespot on ventral hindwing; fv2: anterior eyespot on ventral forewing; hv2: second eyespot on ventral hindwing; jct: distance from junction of the two veins Cu1a and M3 to distal band; fd5: posterior eyespot on dorsal forewing. All morphological traits were divided by wing size.

In the third step, we assessed the level of polyphenism of a selection of secondary sexually-selected traits from experiments A and B: the area of fd5 pupil eyespot divided by wing size, fd5 relative brightness, and the MSP component amounts and ratios (MSP2/MSP1, MSP1/MSP3, MSP2/MSP3). First, we explored the effects of the developmental temperature (continuous variable), sex and their interaction (only in visual sexually-selected traits) on these traits in 8-day old males (experiment A) using polynomial regressions with a quadratic term for developmental temperature. Second, we investigated the variation in MSP composition (i.e. in each component and ratio) throughout adult lifetime using 3, 7, 14, and 21 and 28-days old virgin males (experiment B) in interaction with the rearing temperature of 20°C and 27°C in orthogonal polynomial regressions by including a quadratic term for age. Sample sizes per sex and developmental temperature ranged from 24 to 37 individuals in experiment A and from 4 to 12 individuals in experiment B (Tables 1 and 2).

**Table 2:** Descriptive statistics of the sexually-selected traits per male age and rearing temperature (20°C and 27°C) in experiment B obtained from Nieberding *et al*. [45]’s database.

		3 days		7 days		14 days		21 days		28 days	
Rearing temperatures	Sexual traits	mean±1SD	N	mean±1SD	N	mean±1SD	N	mean±1SD	N	mean±1SD	N
DS-20/20	MSP1 (ng/individual)	545.68±179.48	9	1684.31±334.77	9	3199.04±764.15	9	-	-	2109.44±1155.61	9
	MSP2 (ng/individual)	50.91±61.90	9	242.24±110.07	9	385.73±206.95	9	-	-	465.71±140.78	9
	MSP3 (ng/individual)	1688.72±687.04	9	6748.46±1090.66	9	15232.99±3743.53	9	-	-	9884.90±5617.35	9
	MSP2/MSP1	0.11±0.14	9	0.15±0.09	9	0.12±0.06	9	-	-	0.41±0.42	9
	MSP2/MSP3	0.03±0.04	9	0.04±0.02	9	0.03±0.01	9	-	-	0.12±0.16	9
	MSP1/MSP3	0.36±0.18	9	0.25±0.04	9	0.21±0.03	9	-	-	0.24±0.06	9
WS-27/27	MSP1 (ng/individual)	1089.35±751.89	9	2309.27±1093.92	10	992.70±484.88	12	732.54±375.95	12	-	-
	MSP2 (ng/individual)	208.69±182.95	9	265.23±197.52	10	350.31±126.10	12	423.23±206.85	12	-	-
	MSP3 (ng/individual)	4519.93±2125.92	9	9757.65±5624.66	10	3337.43±1893.51	12	1545.17±1387.58	12	-	-
	MSP2/MSP1	0.27±0.23	9	0.15±0.14	10	0.42±0.21	12	0.74±0.53	12	-	-
	MSP2/MSP3	0.05±0.03	9	0.05±0.06	10	0.15±0.11	12	0.49±0.38	12	-	-
	MSP1/MSP3	0.23±0.05	8	0.27±0.08	10	0.34±0.12	12	0.55±0.20	10	-	-

DS-20/20, WS-27/27: Dry and Wet Season phenotypes reared all their life at 20°C and 27°C, respectively. MSP: Male Sex Pheromone; SD: Standard Deviation; N: sample size

In the fourth step, we tested if the sexually-selected traits (pupil area/wing size, relative brightness, and MSP composition) provide redundant or additional information to morphological wing traits, thus helping to precisely predict the developmental temperature of individuals. We first extracted the residual variance of each sexually-selected trait linearly regressed on hv5 and jct traits, after removing the collinear traits fv2 and hv2 based on variance inflation factor VIF<2 [57]; R package regclass). Second, we used the residuals as response variable to test the effect of the developmental temperature in interaction with sex (when available) in orthogonal polynomial or linear regressions. For these residuals analyses, the sample sizes were of 103 females (DS-17/27 = 30, IS-21/27 and IS23/27 = 25, WS-27/27 = 23), 104 males for visual secondary sexually-selected traits (DS-17/27 and IS-21/27 = 27, IS23/27 = 22, WS-27/27 = 28) and 100 males for chemical secondary sexually-selected traits (DS-17/27 = 27, IS-21/27 = 25, IS23/27 and WS-27/27 = 24).

We ensured the assumptions of normality and homogeneity of variance were fulfilled in scaling, Box-Cox transforming, and centring the response variable using the R package caret in all analyses. When normality could not be reached, outliers were identified, as values under and above boundaries determined respectively by these formula: “1^st^ quartile– 1.5*interquartile range” and “3^rd^ quartile + 1.5* interquartile range” where interquartile range corresponds to “3^rd^ quartile– 1^st^ quartile” [58]. We decided to remove these values when they biased the models toward fits not representative of the whole dataset. Our selection between linear or quadratic models was based on the smallest “Akaike Information Criterion corrected for small sample size” value (AICc; package AICcmodavg) [59]. We applied to full models a stepwise backward selection procedure on two-way interactions. We kept the model with the best fit based on the log-likelihood ratio test and report F-test of overall significance, degrees of freedom and *p*-value. In case of significant interactions in analysis without quadratic term, we assessed whether the slopes of each sex (experiment A) or each rearing temperature (experiment B) were significantly different from zero (package multcomp).

#### 2. Robustness of the quantification of wing morphological traits

We obtained high repeatability estimates (*R* following [60]; and its standard error, s.e., following [61] for all wing morphological and chemical traits based on three independent, repeated measurements per individual using a mixed pool of ca. 30 males and females from different seasonal forms (fv2: *F*_30,62_ = 7873.60, *P*<0.001, *R* = 1.00, s.e. = 1.2e-4; hv2: *F*_29,60_ = 3620.86, *P*<0.001, *R* = 1.00, s.e. = 2.64e-4; hv5: *F*_31,64_ = 16933.39, *P*<0.001, *R* = 1.00, s.e. = 5.48e-05; jct: *F*_31,64_ = 48.73, *P*<0.001, *R* = 0.95, s.e. = 0.02; forewing size: *F*_31,64_ = 42073.17, *P*<0.001, *R* = 1.00, s.e. = 2.21e-5; hindwing size: *F*_31,64_ = 1069250, *P*<0.001, *R* = 1.00, s.e. = 8.68e-7; fd5 pupil area: *F*_30,62_ = 32.63, *P*<0.001, *R* = 0.91, s.e. = 0.03; fd5 pupil relative brightness: *F*_30,62_ = 75.84, *P*<0.001, *R* = 0.96, s.e. = 0.01). We made sure that the compressed format pictures (.jpg) we used to measure the relative brightness of fd5 pupils produced comparable results to the pupil relative brightness extracted from the corresponding RAW format pictures (paired *t*-test *t*_30_ = 1.4, *P* = 0.2; following [52]). Finally, we confirmed that our camera settings and picture processing methodology accurately quantified UV reflectance [51,52], as follows: the mean gray values of a small area (10373 pixels) measured on each of the four reflection standard blocs in a sub-sample of 10 pictures converted in brightness stack with ImageJ strongly and positively correlate with their known UV reflectance (10/25/50/95%) (Spearman’s rank correlation: r_s_ = 0.90, *p*<0.001, S = 1058, n = 40). In other words, the measured relative brightness of pupils gives a ranking comparable to the one of their UV reflectance.

## Results

### Polyphenism of wing morphological traits (hv2, fv2, hv5 and jct) including sexually selected traits (fd5) for discriminating between seasonal forms

**Fig 3:**
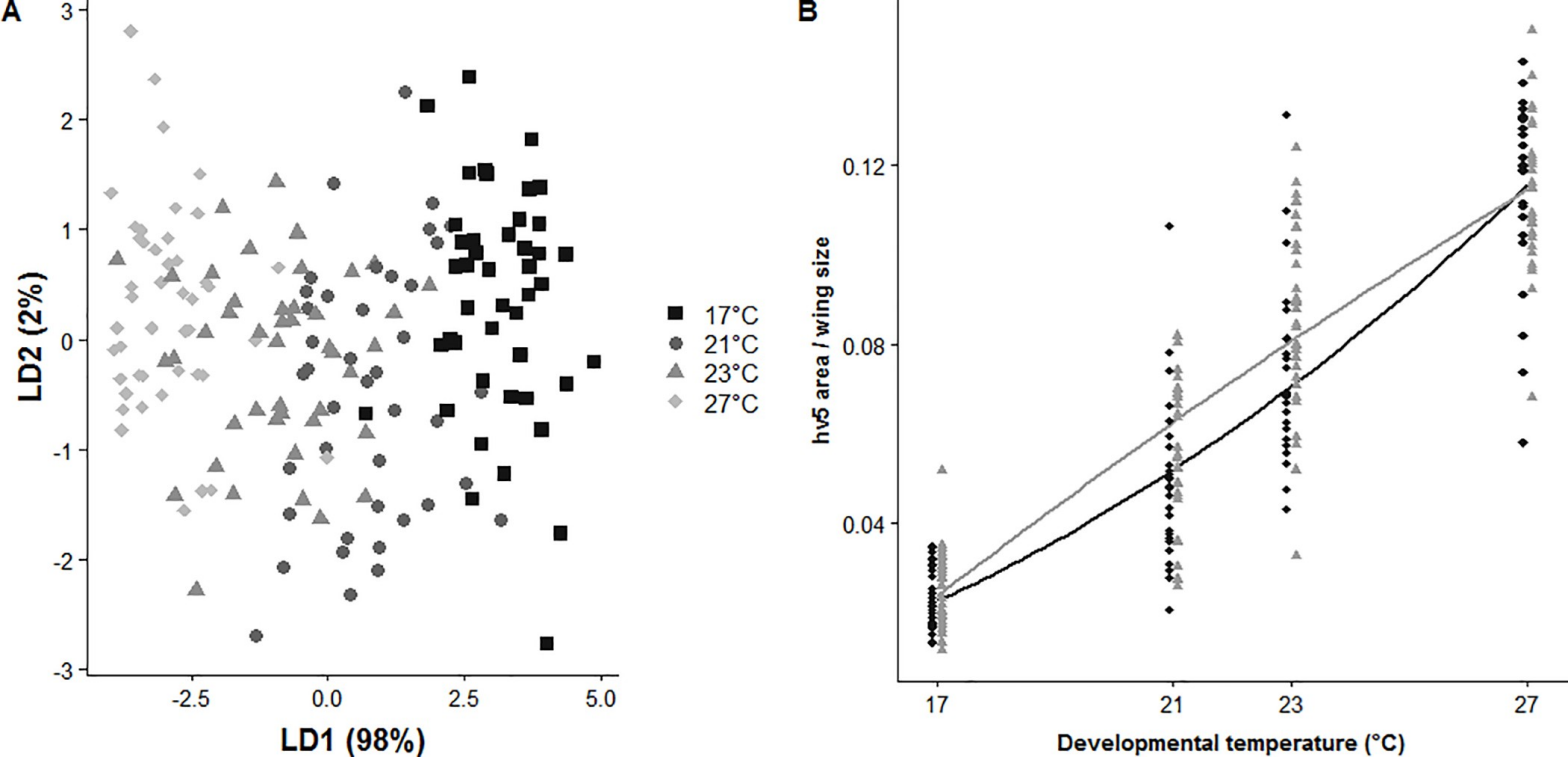
Characterization of the most polyphenic traits. (A) The first two Linear Discriminants (LD1 and LD2) from the Linear Discriminant Analysis of the polyphenic, wing morphological traits (hv5, hv2, and fv2 areas, and jct, each divided by wing size) and the visual sexually-selected traits (fd5 area divided by wing size and fd5 relative brightness) in both sexes across developmental temperatures (square: 17°C, disk: 21°C, triangle: 23°C, diamond: 27°C combined with decreasing darkness). (B) Area of hv5 eyespots divided by wing size (mm^2^) for both sexes (males in grey triangle and females in black diamonds) across developmental temperatures.

Here we examined the following morphological wing traits (i.e. hv5, hv2, jct and fv2) and two visual sexually-selected traits (area and relative brightness of fd5 pupil). Most of these traits displayed sexual dimorphism (hv5, hv2, jct, not fv2), but we reported results combining both sexes because sexes varied often similarly in response to developmental temperature (see details below). The areas of fv2, hv2, and hv5 eyespots (corrected by wing size) were strongly and positively correlated to each other, and all weakly and negatively correlated to jct (hv2-fv2: *r*_s_ = 0.94; fv2-hv5: *r*_s_ = 0.90; fv2-jct: *r*_s_ = -0.46; hv2-hv5: *r*_s_ = 0.93; hv2-jct: *r*_s_ = -0.44; hv5-jct: *r*_s_ = -0.47; all *P*<0.001). Regarding visual sexually-selected traits, fd5 area correlated positively to fd5 relative brightness (*r*_s_ = 0.29, *P*<0.001), but was not correlated to any of the four wing morphological traits (fv2-fd5 area: *r*_s_ = 0.69, *P* = 0.36; jct-fd5 area: *r*_s_ = -0.052, *P* = 0.49; hv5-fd5 area: *r*_s_ = 0.14, *P* = 0.07; hv2-fd5 area: *r*_s_ = -0.01, *P* = 0.90). Fd5 relative brightness correlated negatively to fv2, hv5 and hv2 (fv2-fd5 relative brightness: *r*_s_ = -0.23, *P* = 0.003; hv5-fd5 relative brightness: *r*_s_ = -0.17, *P* = 0.02; hv2-fd5 relative brightness: *r*_s_ = -0.28, *P*<0.001), but was not correlated to jct (*r*_s_ = -0.06, *P* = 0.43). A discriminant analysis revealed that 98% of the variance generated by the four developmental temperatures was explained by the first axis, best represented by hv5 area (linear discriminant coefficient: hv5 = -1.52, hv2 = -0.81, fv2 = -0.11, jct = 0.02, fd5 area = -0.33, fd5 relative brightness = 0.08; Fig 3A).

### Extent of polyphenic variation of the four wing morphological traits (hv2, fv2, hv5 and jct)

Using univariate analyses, we found that eyespot areas corrected by wing size increased with developmental temperatures in both sexes (hv5: *F*_3,216_ = 302; hv2: *F*_2,217_ = 414.9; fv2: *F*_2,217_ = 324.3; all *P*<0.001; Fig 3B; S1A and S1B Fig; Table 1, summary of models in S2 Table). Hv5, hv2 and fv2 displayed sexual dimorphism with males producing larger eyespots than females (Fig 3B; S1A and S1B Fig). Finally, jct length decreased with developmental temperature (*F*_5,214_ = 9.02, *P*<0.001), but with a steeper decline in females than in males (significant developmental temperature*sex interaction; S1C Fig).

### Extent of polyphenic variation of secondary sexually-selected wing traits

**Fig 4:**
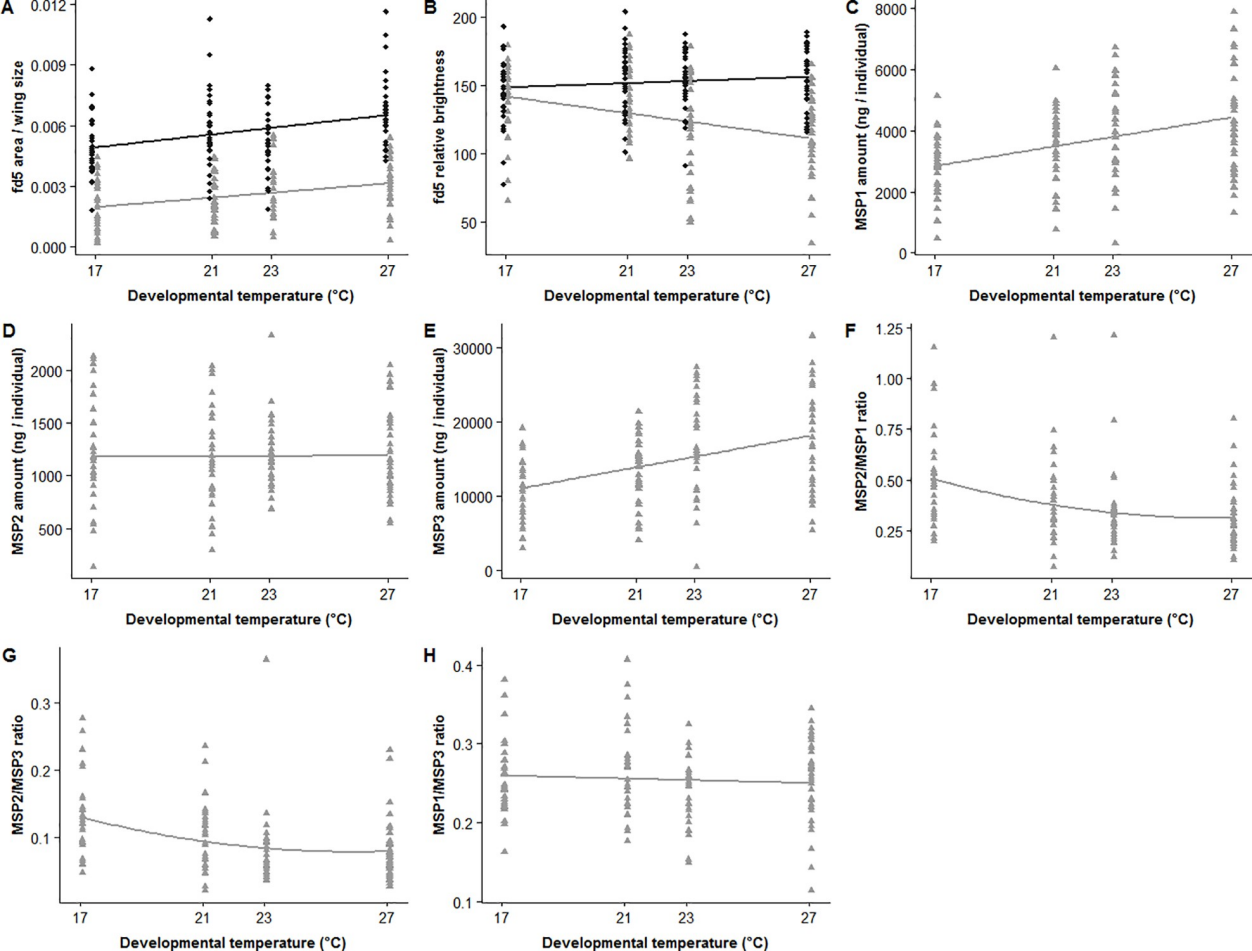
Variation in secondary sexually-selected traits as a function of the developmental temperature (°C) experienced by 8-day old males (grey triangles) and females (black diamonds) in experiment A. (A) fd5 area (mm^2^)/wing size (mm^2^); (B) fd5 relative brightness, (C) MSP1, (D) MSP2 and (E) MSP3 amounts (ng/individual), (F) MSP2/MSP1, (G) MSP2/MSP3 and (H) MSP1/MSP3 ratios. Lines represent the model estimates that best fit the data.

The pupil area of the fd5 dorsal forewing eyespot divided by wing size increased from the lowest (17°C) to the highest (27°C) developmental temperature in both sexes, but displayed sexual dimorphism, being larger in females than in males (*F*_2,229_ = 137.3, *P*<0.001; Fig 4A; Table 1; S2 Table). Its relative brightness decreased with increasing temperatures in males while it remained stable, and thus higher at high developmental temperatures in females (developmental temperature*sex interaction: *F*_3,253_ = 26.1, *P*<0.001; linear function slope differed from zero in males: *t* = -4.30, *P*<0.001, not in females: *t* = 1.24, *P* = 0.38; Fig 4B; Table 1, S2 Table).

In 8-day old males, MSP1 and MSP3 amounts increased linearly with developmental temperature (MSP1: *F*_1,130_ = 21.98; MSP3: *F*_1,130_ = 26.6; both *P*<0.001; Fig 4C and 4E; Table 1; S2 Table), while MSP2 amounts remained stable (*F*_1,130_ = 0.003; *P* = 0.96; Fig 4D; Table 1; S2 Table). MSP2/MSP1 and MSP2/MSP3 ratios decreased from 17 to 23°C and then stabilized up to 27°C (MSP2/MSP1: *F*_2,127_ = 10.26; MSP2/MSP3: *F*_2,127_ = 11.24; both *P*<0.001; Fig 4F and 4G; Table 1; S2 Table) whereas MSP1/MSP3 ratio did not vary with the developmental temperature (*F*_1,1308_ = 0.19; *P* = 0.66; Fig 4H; Table 1; S2 Table).

**Fig 5:**
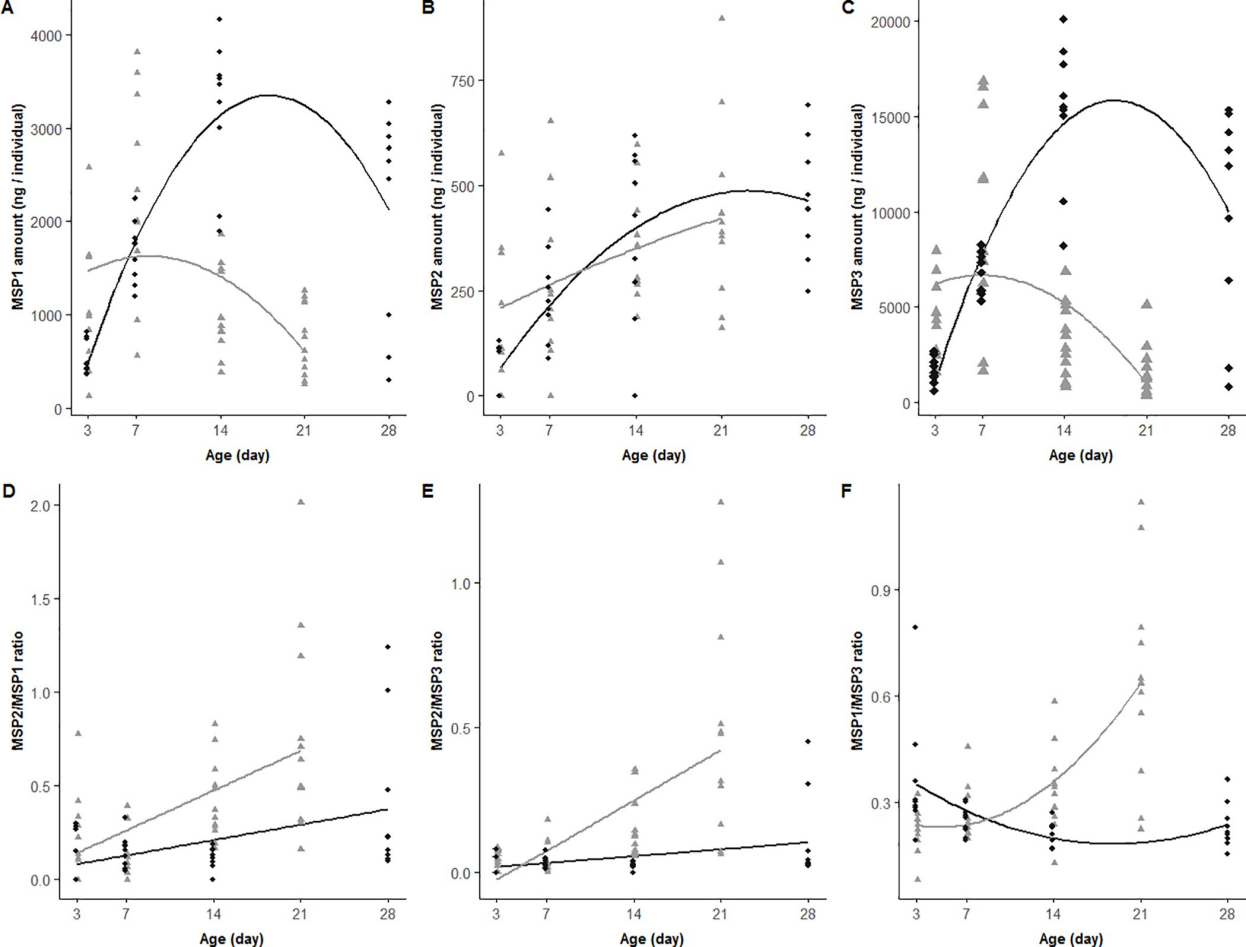
Variation in MSP composition as a function of the age of males reared at 20°C (black diamonds) and at 27°C (grey triangles) in experiment B. (A) MSP1, (B) MSP2 and (C) MSP3 amounts (ng/individual), (D) MSP2/MSP1, (E) MSP2/MSP3 and (F) MSP1/MSP3 ratios. Lines represent the model estimates that best fit the data.

When MSP composition was sampled throughout male adult life, MSP2 amounts increased with age similarly at the two rearing temperatures of 20°C and 27°C (*F*_3,75_ = 15.36, *P*<0.001; Fig 5B; Table 2; S3 Table). In contrast, MSP1 and MSP3 amounts, and all MSP ratios, changed differently throughout male lifespan depending on rearing temperatures (temperature*age interaction; MSP1: *F*_5,73_ = 15.55; MSP3: *F*_5,73_ = 22.47; MSP2/MSP1: *F*_3,75_ = 15.17; MSP2/MSP3: *F*_3,75_ = 30.53; MSP1/MSP3: *F*_5,70_ = 11.25; all *P*<0.001; Fig 5A, 5C-5F; Table 2; S3 Table). Both MSP1 and MSP3 amounts showed a polynomial relationship, which peaked at 7 days for males reared at 27°C and at 14 days for those reared at 20°C. In addition, males reared at 27°C, as compared to those reared at 20°C, had higher amounts at 3 days and lower ones at 14 and 28 days. All MSP ratios steeply increased with age in males reared at 27°C but in a slower way for males reared at 20°C for MSP2/MSP1 and MSP2/MSP3 ratios. MSP1/MSP3 ratio tended to decrease across age. As a consequence, MSP1/MSP3 was lower in 3-day old males and all ratios were higher in 14- and 28-day old males at 27°C as compared to 20°C (Fig 5; Table 2; S3 Table).

### Comparing polyphenism of wing morphological and sexually-selected traits

The developmental temperature in interaction with sex explained a substantial variability in visual, but not in chemical, secondary sexually-selected traits after controlling for variation in the wing morphological traits (i.e. using residuals on hv5 and jct) in experiment A (S2 Fig; S2 Table). Similarly to the uncorrected values of visual secondary sexually-selected traits, sexual dimorphism remained as females had larger area residuals (*F*_2,204_ = 117.4, *P*<0.001) and higher relative brightness residuals (*F*_5,201_ = 10.85, *P*<0.001) for their fd5 pupil than males across developmental temperatures (S2A and S2B Fig). In addition, the residuals of relative brightness showed a significant interaction between sexes and temperature: residuals decreased in males with increasing developmental temperatures (similarly to the uncorrected values) while it increased up to 21°C to stabilize until 27°C in females (S2B Fig).

In contrast, the male-specific, chemical secondary sexually-selected traits showed redundancy with the polyphenic wing morphological traits (hv5 and jct), as the residuals of MSP amounts and ratios on these traits did not vary with developmental temperatures (MSP1: *F*_1,98_ = 0.07, *P* = 0.80, MSP2: *F*_2,97_ = 1.62, *P* = 0.20, MSP3: *F*_1,98_ = 0.005, *P* = 0.95; MSP2/MSP1: *F*_2,97_ = 1.93, *P* = 0.15; MSP2/MSP3: *F*_2,97_ = 2.88, *P* = 0.06; MSP1/MSP3: *F*_1,98_ = 0.71, *P* = 0.40; S2C–S2H Fig; S2 Table).

## Discussion

The aims of our study were three-fold. First, we identified the most polyphenic morphological wing trait to characterize seasonal phenotypes among those usually used in previous studies on *B*. *anynana*. We found that the area of the fifth eyespot on ventral hindwings corrected by wing size increased with developmental temperatures and allowed the best discrimination in both sexes between four developmental temperatures typical of dry (17°C), intermediate (21°C and 23°C) and wet (27°C) seasons in the wild. The use of this trait will ease and improve the accuracy of polyphenism quantification in future studies. Second, we found that individuals reared at intermediate temperatures show intermediate trait values in this species for a suite of traits that evolve either by natural or sexual selection. This had been shown for several morphological wing traits but not for many sexually selected ones [21,30,45,48,62]. We found that in both sexes, pupil area of the fd5 dorsal forewing eyespot increased with developmental temperatures; its relative brightness decreased in males with increasing temperature, while it remained stable in females; females had overall UV-brighter and larger fd5 eyespot pupils than males. The composition of the male sex pheromone also varied across developmental temperatures: the amounts of MSP1 and MSP3 components increased, and MSP2/MSP1 and MSP2/MSP3 ratios decreased with increasing temperature, but MSP2 amounts and MSP1/MSP3 ratio remained stable. Interestingly, the visual and chemical sexually-selected traits provided respectively additional and redundant information to the polyphenic wing morphological traits, which may improve the assessment of sexual partners in relation to their thermal history. Third, we found significant age-related changes in MSP production in both WS and DS forms: the directions of MSP developmental reaction norms reversed with increasing age, such that at an older age DS individuals produce more MSP1 and MSP3 than WS ones.

Assessing the developmental temperature of sexual partners with accuracy can be relevant to fitness. Indeed, mating with DS males increases female longevity and egg production at least in laboratory settings [23]. Hence, mate discrimination in favour of DS phenotypes may improve female fitness. More specifically, mating with the IS phenotype provides unknown fitness effects, but is *a priori* considered as maladaptive because this phenotype may not be adapted either to dry or to wet seasons compared to extreme phenotypes [26]. Sexually-selected traits that differ between WS, DS and IS phenotypes may thus act as additional cues decreasing the investment in time to evaluate mate quality [28,29], and helping to discriminate the IS phenotype.

Here we found that the variation in visual (fd5 pupil area) and chemical (MSP1, MSP3, MSP2 ratios) secondary sexually-selected traits may inform potential mating partners about the thermal environment males and females experienced during development. Importantly, while the polyphenic chemical sexually-selected traits are not more informative than the polyphenic wing morphological traits (hv5, fv2, hv2 and jct), the sexually-selected trait “relative brightness of fd5 pupils” provides additional discrimination power compared to hv5, fv2, hv2 and jct traits about the developmental temperature history of individuals. This is a novel finding showing how multiple polyphenic wing traits can provide either redundant or additional discriminative information about developmental history in *B*. *anynana* (S1 Table). Of note, individuals developing at 21°C (IS-21/27 treatment) appeared morphologically and physiologically more similar to the DS form (DS-17/27 treatment) while individuals developing at 23°C (IS-23/27 treatment) were phenotypically closer to the WS form.

Previous studies quantified the same sexually-selected traits in WS and DS forms for MSP composition: [43,45]. We did not directly compare MSP titres across experiment A and B because there are many unknown (co-) variables to consider. Indeed, these experiments A and B were conducted many years apart and in different laboratories. Furthermore, experiments A and B only have one overlapping thermal treatment (WS-27/27). Regarding MSP1 and MSP3 components, our results are rather congruent with the ones of Dion *et al*. [43] (Table 3). We similarly found that 8-day old DS males (DS-17/27) produced lower amounts of MSP1 and MSP3 components than WS males (WS-27/27) while the amount of MSP2 component was similar between DS and WS males in our experiment A (Table 3), as in Dion *et al*. [43].

**Table 3:** Comparison of sexually-selected traits between dry and wet season phenotypes using this and other studies.

Traits	Thermal treatments	Experiment A	Experiment B [45]	Dion *et al*. [43]	Prudic *et al*. [23]	Everett *et al*. [63]	Bergen *et al*. [62]	Mateus *et al*. [30]
fd5 area	♂ DS-17/17	-	-	-	0.056±0.032^a^	-	^-^	-
	♂ DS-17/27	2.00e-3±1.26e-3^b^	-	-	-	-	-	-
	♂ DS-19/19	-	0.26±0.14^a^	-	-	-	-	-
	♂ IS-21/21	-	-	-	-	-	0.0014±0.00082^b^	-
	♂WS-27/27	3.12e-3±1.36e-3^b^	-	-	0.111±0.081^a^	-	0.0021±0.0010^b^	-
	♂WS-28/28	-	0.38±0.16^a^	-	-	-	-	-
	♀ DS-17/17		-	-	0.310±0.081^a^	-	^-^	-
	♀ DS-17/27	4.97e-3±1.44e-3^b^	-	-	-	-	-	-
	♀ IS-21/21	-	-	-	-	-	0.0039±0.0009^b^	-
	♀ DS-19/19	-	-	-	-	-	-	0.00020±0.00010^b^
	♀WS-27/27	6.74e-3±1.77e-3^b^	-	-	0.310±0.065^a^	-	0.0045±0.0011^b^	0.00031±0.000087^b^
fd5 relative brightness	♂ DS-17/17	-	-	-	47±6.45^d^	57.5^d^^e^	-	-
	♂ DS-17/27	139.51±25.65^c^	-	-	-	-	-	-
	♂WS-27/27	112.55±29.74^c^	-	-	58±9.68^d^	56^d^^e^	-	-
	♀ DS-17/17	-	-	-	56±6.45^d^	55^d^^e^	-	-
	♀ DS-17/27	143.71±24.86^c^	-	-	-	-	-	-
	♀WS-27/27	152.36±23.20^c^	-	-	50±4.84^d^	46^d^^e^	-	-
MSP1	DS-17/17	-	-	920.3±389.72	-	-	-	-
	DS-17/27	2794.66±1051.70	-	1370.83±445.83	-	-	-	-
	DS-20/20	-	1684.31±334.77	-	-	-	-	-
	WS-27/27	4337.01±1675.47	2309.27±1093.92	3691.96±1613.96	-	-	-	-
MSP2	DS-17/17	-	-	392.16±220.22	-	-	-	-
	DS-17/27	1216.26±495.74	-	800.95±396.48	-	-	-	-
	DS-20/20	-	242.24±110.07	-	-	-	-	-
	WS-27/27	1204.18±409.20	265.23±197.52	736.63±501.38	-	-	-	-
MSP3	DS-17/17	-	-	3761.86±1047.37	-	-	-	-
	DS-17/27	10988.28±4313.78	-	7513.83±1324.41	-	-	-	-
	DS-20/20	-	6748.46±1090.66	-	-	-	-	-
	WS-27/27	17657.83±6959.18	9757.65±5624.66	16367.28±4314.27	-	-	-	-

For fd5 pupil area, measurements are presented

^a^in mm^2^ and

^b^corrected by squared wing size (mm^2^). Fd5 pupil relative brightness measurements are

^c^unitless (grayscale values from 0 to 255) or

^d^in % of UV reflectance. Pheromone components (MSP1, MSP2 and MSP3) are in ng/individuals. All values are given as mean ± SD

^e^except when only approximate average values were available.

It is important to consider adult flexibility of secondary sexually-selected traits in *B*. *anynana* because this species lives for months in the natural environment (3 months for the WS form and 6 months for the DS form), and DS males are expected to mate mostly at the end of the dry season [7,27]. Hence, sexual selection may shape secondary sexual traits at ages that are not usually studied [35,44]. Our quantification of MSP components throughout male lifetime in DS-20/20 and WS-27/27 males (experiment B) revealed that the developmental reaction norms (for MSP1 and MSP3) reversed with increasing age between seasonal forms, such that at an older age (from 14-day old) DS individuals produce more MSP than WS ones. The change in amounts of MSP1 and MSP3 components across ages is consistent with the one observed earlier in WS males only [35]. We propose that the slower rate of MSP1 and MSP3 increase in DS-20/20 compared to WS-27/27 males is due to the lower resting metabolic rate of the former when individuals are maintained under their respective thermal treatment [19,21]. In contrast, MSP2 amount kept increasing across ages in both seasonal forms as previously observed in WS-27/27 [35]. Importantly, stable amounts of MSP2 component across developmental temperatures in our two experiments suggest that hexadecanal may matter for mating success for all males independently of their developmental history, extending the importance of the MSP2 component for mating success, which is so far established for WS-27/27 males [35,36,64,65]. Our results highlight the complexity of how sexual selection shapes the male sex pheromone composition in this polyphenic species that displays a sex role reversal across seasons [23,43]. Of note, our experimental design does not allow us to disentangle whether the temperature experienced at the larval or adult stage, or both, are responsible for differences in MSP composition. However, the temperature at larval stage is supposed to forecast the seasonal environment the adult will have to face in nature [14], meaning that thermic environment of the larva and adults are usually correlated in the wild [27,66].

Regarding the sexually-selected fd5 eyespot on the forewing, the size of male pupil area increased with increasing developmental temperature, such that WS males had larger pupils than DS males as previously documented [23,45]. Contradictory results were observed in published literature concerning female pupil area and UV reflectance in both sexes (Table 3). The area of female pupils increased with developmental temperature in our study as in Bergen *et al*. [62], but it remained stable in other studies [23,30]. UV reflectance of female pupils was stable across developmental temperatures in experiment A and in Everett *et al*. [63], but it decreased with developmental temperature in Prudic *et al*. [23]. In males, we found that pupil UV reflectance decreased with increasing developmental temperature such that DS males had brighter pupils than WS ones, but the reverse was observed in other studies [23,63]. Hence, we did not observe changes in investment of visual sexually-selected traits caused by a seasonal reversal in sexual roles [23]. Although others used a spectrophotometer [23,63] whereas we (as [32]) used a UV-sensitive camera to measure UV reflectance, it is unlikely that methodological differences explain these discrepancies because the UV reflectance obtained from spectrophotometers correlates positively to values obtained from pictures [67]; please see our Materials and Methods for testing this assumption in our dataset). Hence, measurements of relatively higher or lower UV reflectance of fd5 pupils between sexes and developmental temperatures is unlikely to depend on the method (spectrophotometer or camera) used for quantifying changes. One possible explanation for these discrepancies between published literature and our results could be due to differences in the time period of thermal treatments among studies. Indeed, our thermal treatment occurred between the fourth larval instar and the first half of pupation whereas the ones in Prudic *et al*. [23] and Everett *et al*. [63] lasted from the egg to adult stage. Different critical developmental windows for wing patterns and behaviours have already been reported [7,18,20,21], and we may have missed the developmental period sensitive to temperature for some wing structures responsible for the UV reflectance of fd5 pupils. Importantly, we used three times larger sample sizes and a more robust statistical approach providing higher confidence in the conclusions drawn from our results of UV reflectance than some previously published estimates. We thus suggest that larger sample sizes in this study compared to Prudic *et al*. [23] and Everett *et al*. [63] could be another explanation to these discrepancies.

*B*. *anynana* is considered as a polyphenic species in the scientific community [5,6,19]. However, hv5, hv2 and fv2 reaction norms obtained with our four thermic treatments were linear and did not show a binomial relationship like in other polyphenic species [5,6]. This may be due to the artificial (stable temperature throughout development) and narrow (17°C to 27°C) temperature treatments classically applied in the laboratory and for this experiment. Indeed, we focused on the IS phenotype and omitted effect temperatures on traits under 17°C and above 27°C whereas in nature, this species can undergo a range temperatures from 10°C to 29°C with circadian and seasonal thermic variations that our experimental design does not provide [7]. Hence, we cannot reject the term “polyphenic” associated to *B*. *anynana* although the traits we quantified had a linear reaction norm.

## Conclusions

Our study brings three novel aspects about the model species, *B*. *anynana*. First, we found that the fifth eyespot on ventral hindwing is the most polyphenic trait that can be used to ease the quantification of polyphenism in future studies. Second, we showed that naturally and sexually selected trait values changed following a continuous relationship across developmental temperatures producing linear reaction norms, except for MSP2 amount. Our findings suggest that females could use these sexually-selected traits for assessing the developmental history of IS males. Third, we focused on the plasticity of the chemical sexually-selected trait, MSP composition, across adult lifespan and we pointed out that patterns of MSP composition are reversed in young, compared to older males, across developmental temperatures. Our results illustrate the complexity of sexual selection effects on sexually-selected traits, particularly when one takes the long lifetime of a species (several months in the field for *B*. *anynana*) into account in the experimental design. We suggest that the stable expression of MSP2 amounts across developmental temperatures may be due to directional sexual selection on this trait, which is central to mating success, in all seasonal forms. Further work will be needed to demonstrate the role of MSP2 amount in mating success of DS and IS males.

The selective pressure and fitness associated to the IS phenotype in *B*. *anynana* is currently unknown. Their low density across the year compared to DS and WS forms (see introduction) suggests that the IS phenotype is selected against during a major part of the year [27], which is characteristic of polyphenic species [5,26]. However, the IS phenotype becomes the most abundant phenotype during seasonal transitions, which suggests that this phenotype may be associated either with some fitness benefits in the field or that the IS phenotype results from developmental constraints [7,27]. In this regard, our finding that several polyphenic traits display linear reaction norms concurs these suggestions about the IS phenotype, at least in some, transitional environments.

## Supporting information

S1 Fig Variation in wing morphological traits in both sexes across developmental temperatures (°C) in experiment A. A: hv2 area (mm^2^)/wing size (mm^2^); B: fv2 area (mm^2^)/wing size (mm^2^); C: jct length (mm)/wing size (mm)). 8-day old males in grey triangles and females in black diamonds. Lines represent the model estimates that best fit the data.
(TIFF)

S2 Fig Variation in residuals of secondary sexually-selected traits on polyphenic wing morphological traits (hv5 area and jct) across developmental temperatures (°C) in both sexes in experiment A. Visual sexually-selected trait residuals corresponded to (A) fd5 area (mm^2^)/wing size (mm^2^), and (B) fd5 relative brightness, and chemical sexually-selected trait residuals to (C) MSP1, (D) MSP2 and (E) MSP3 amounts (ng/individual), (F) MSP2/MSP1, (G) MSP2/MSP3, and (H) MSP1/MSP3 ratios. 8-day old males are represented in grey triangles and females in black diamonds. Lines represent the model estimates that best fit the data.
(TIFF)

S1 Table Review of the wing traits reported to be polyphenic by 13 studies published between 1993 and 2017. (DOCX)

S2 Table Summary of models testing for the changes in wing morphological traits, in secondary sexually-selected traits, and in secondary sexually-selected traits as residuals on polyphenic wing morphological traits across the four developmental temperatures (17°C, 21°C, 23°C, 27°C) in experiment A. (DOCX)

S3 Table Summary of models testing for the changes in chemical secondary sexually-selected traits throughout male adult life, i.e. from 3 to 28 days at the rearing temperature of 20°C, and from 3 to 21 days at 27°C, in experiment B. (DOCX)

S1 File Experiment A dataset presenting wing morphological and chemical trait values across four developmental temperatures per individual. (XLSX)

S2 File Experiment B dataset presenting male sex pheromone (MSP) component amounts across different ages and two developmental temperatures per individual. (XLSX)
