# Supplementary figures and images for: Polyphenism of visual and chemical secondary sexually-selected wing traits in the butterfly Bicyclus anynana: How different is the intermediate phenotype?
Source: PLoS One. 2019 Nov 18;14(11):e0225003. doi: 10.1371/journal.pone.0225003 (PMC6860419; doi:10.1371/journal.pone.0225003)

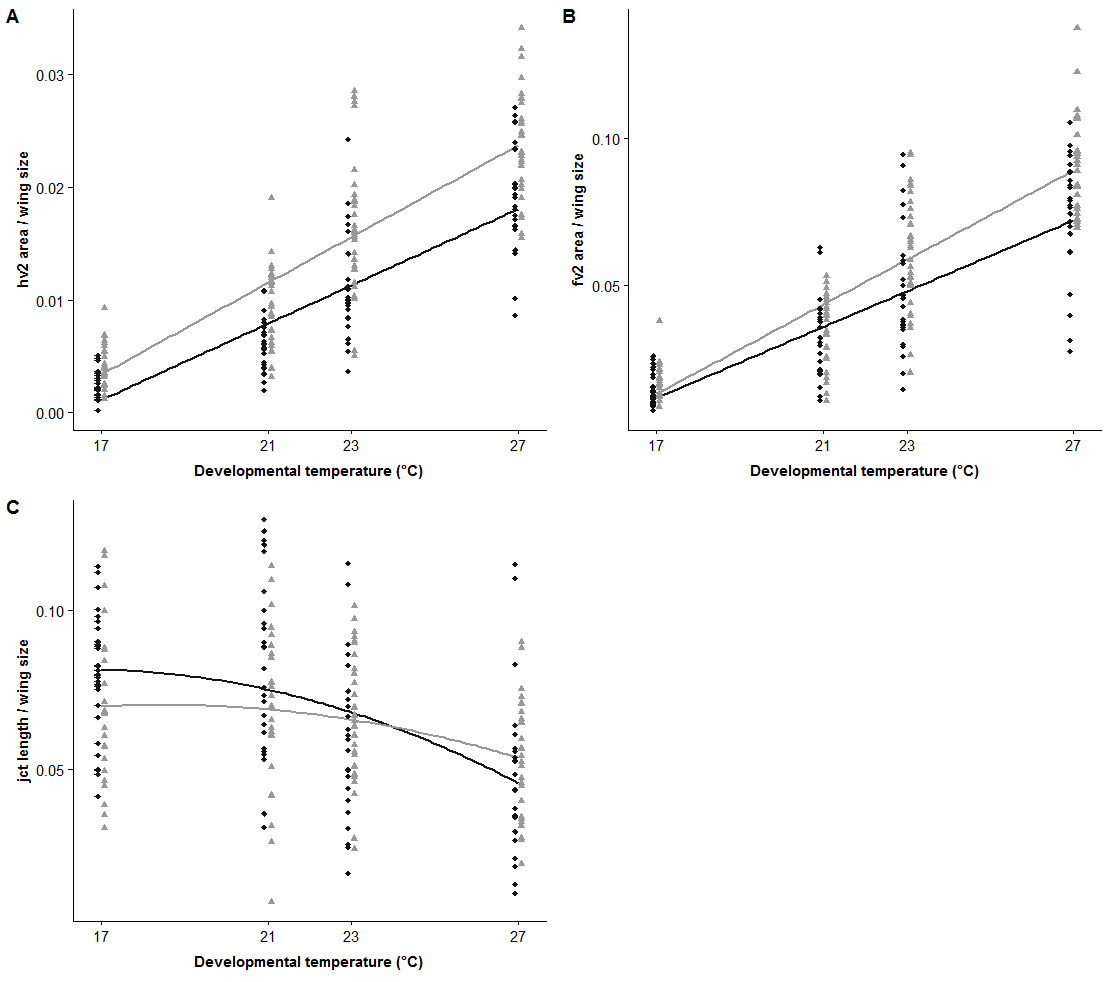

Supplement: S1 Fig — A: hv2 area (mm2)/wing size (mm2); B: fv2 area (mm2)/wing size (mm2); C: jct length (mm)/wing size (mm)). 8-day old males in grey triangles and females in black diamonds. Lines represent the model estimates that best fit the data. (TIFF) [file pone.0225003.s001.tiff]

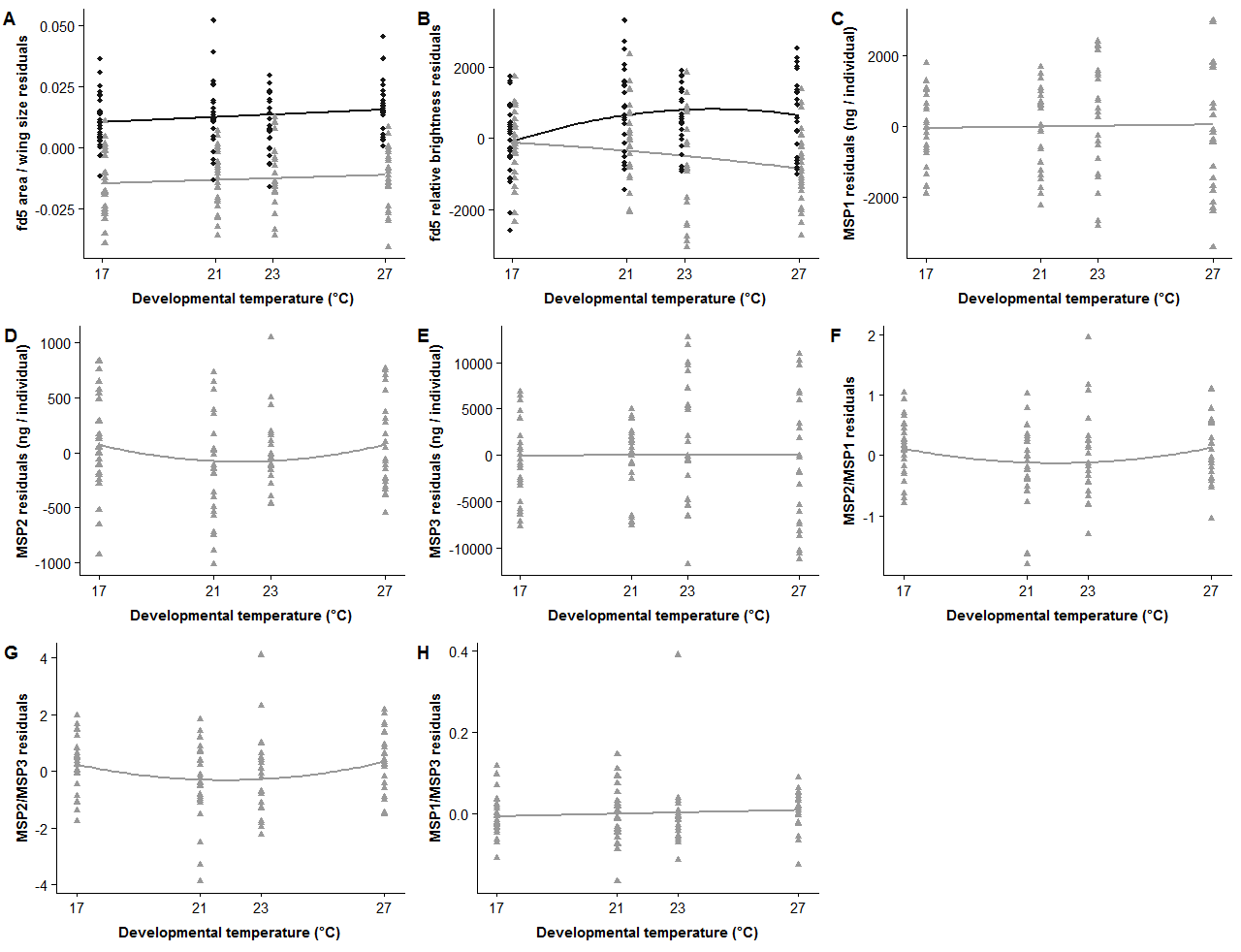

Supplement: S2 Fig — Visual sexually-selected trait residuals corresponded to (A) fd5 area (mm2)/wing size (mm2), and (B) fd5 relative brightness, and chemical sexually-selected trait residuals to (C) MSP1, (D) MSP2 and (E) MSP3 amounts (ng/individual), (F) MSP2/MSP1, (G) MSP2/MSP3, and (H) MSP1/MSP3 ratios. 8-day old males are represented in grey triangles and females in black diamonds. Lines represent the model estimates that best fit the data. (TIFF) [file pone.0225003.s002.tiff]
