# Supplementary material for: Polyphenism of visual and chemical secondary sexually-selected wing traits in the butterfly Bicyclus anynana: How different is the intermediate phenotype?
Source: PLoS One. 2019 Nov 18;14(11):e0225003. doi: 10.1371/journal.pone.0225003 (PMC6860419; doi:10.1371/journal.pone.0225003)
Supplement: S1 Table — (DOCX) [file pone.0225003.s003.docx]

# Supplementary material:

## Supplementary S1 Table

S1 Table. **Review of the wing traits known to be polyphenic in 13 studies published between 1993 and 2017.** The traits we selected are in bold, and were corrected by wing size.

| References  Wing traits | | | Holloway *et al.* [1] | Brakefield & Mazzotta [2] | Roskam & Brakefield [3] | Kooi & Brakefield [4] | Roskam & Brakefield [5] | Wijngaarden & Brakefield [6] | De Jong *et al.* [7] | Oostra *et al.* [8] | Prudic *et al.* [9] | Everett *et al.* [10] | Mateus *et al.* [11] | Oostra *et al.* [12] | Van Bergen *et al.* [13] | Number of appearances |
| --- | --- | --- | --- | --- | --- | --- | --- | --- | --- | --- | --- | --- | --- | --- | --- | --- |
| Ventral forewing | **Anterior eyespot size (fv2)** | | X | X | X | X | X | X |  |  |  |  | X |  | X | 8 |
|  | Posterior eyespot size | | X |  | X |  | X |  |  |  |  |  | X |  | X | 5 |
|  | Chevron: Width marginal band between vein Cu1a and Cu1b | |  |  | X |  |  |  |  |  |  |  |  |  |  | 1 |
|  | Width median band | |  |  | X |  |  |  |  |  |  |  | X |  | X | 3 |
| Ventral hindwing | **2^nd^ eyespot (hv2)** | |  |  | X |  | X |  |  |  |  |  | X | X |  | 4 |
|  | **5^th^ eyespot (hv5)** | | X | X | X | X | X | X | X | X |  |  | X | X | X | 11 |
|  | 4^th^ wing cell | Colour spectrum (Red-Green-Blue: RGB) of the dark area |  |  |  |  |  |  | X |  |  |  |  |  |  | 1 |
|  |  | Colour spectrum (RGB) of the bright area |  |  |  |  |  |  | X |  |  |  |  |  |  | 1 |
|  |  | Dark-bright contrast |  |  |  |  |  |  | X |  |  |  |  |  |  | 1 |
|  | **Width median band (jct)** | |  | X | X | X | X | X | X | X |  |  | X |  | X | 9 |
|  | Chevron: Width marginal band between vein Cu1a and Cu1b | |  |  | X |  |  |  |  |  |  |  |  |  |  | 1 |
| Dorsal forewing | Anterior eyespot size | | X |  | X |  |  |  |  |  | X |  | X |  | X | 5 |
|  | Posterior eyespot size | | X |  | X |  |  |  |  |  | X |  | X |  | X | 5 |
|  | UV reflectance of eyespot | |  |  |  |  |  |  |  |  |  | X |  |  |  | 1 |
| **Wing size** | | |  | X | X |  | X |  | X |  |  |  | X | X | X | 7 |

# Supplementary references

1. Holloway GJ, Brakefield PM, Kofman S. The genetics of wing pattern elements in the polyphenic butterfly, Bicyclus anynana. Hered Lond. 1993;70: 179–179.

2. Brakefield PM, Mazzotta V. Matching field and laboratory environments: effects of neglecting daily temperature variation on insect reaction norms. J Evol Biol. 1995;8: 559–573.

3. Roskam JC, Brakefield PM. A comparison of temperature-induced polyphenism in African Bicylus butterflies from a seasonal savannah-rainforest ecotone. Evolution. 1996; 2360–2372.

4. Kooi RE, Brakefield PM. The critical period for wing pattern induction in the polyphenic tropical butterfly Bicyclus anynana (Satyrinae). J Insect Physiol. 1999;45: 201–212.

5. Roskam JC, Brakefield PM. Seasonal polyphenism in Bicyclus (Lepidoptera: Satyridae) butterflies: different climates need different cues. Biol J Linn Soc. 1999;66: 345–356.

6. Wijngaarden PJ, Brakefield PM. Lack of response to artificial selection on the slope of reaction norms for seasonal polyphenism in the butterfly Bicyclus anynana. Heredity. 2001;87: 410–420.

7. De Jong MA, Kesbeke F, Brakefield PM, Zwaan BJ. Geographic variation in thermal plasticity of life history and wing pattern in Bicyclus anynana. Clim Res. 2010;43: 91.

8. Oostra V, de Jong MA, Invergo BM, Kesbeke F, Wende F, Brakefield PM, et al. Translating environmental gradients into discontinuous reaction norms via hormone signalling in a polyphenic butterfly. Proc R Soc Lond B Biol Sci. 2011;278: 789–797.

9. Prudic KL, Jeon C, Cao H, Monteiro A. Developmental plasticity in sexual roles of butterfly species drives mutual sexual ornamentation. Science. 2011;331: 73–75.

10. Everett A, Tong X, Briscoe AD, Monteiro A. Phenotypic plasticity in opsin expression in a butterfly compound eye complements sex role reversal. BMC Evol Biol. 2012;12: 232. doi:10.1186/1471-2148-12-232

11. Mateus ARA, Marques-Pita M, Oostra V, Lafuente E, Brakefield PM, Zwaan BJ, et al. Adaptive developmental plasticity: Compartmentalized responses to environmental cues and to corresponding internal signals provide phenotypic flexibility. BMC Biol. 2014;12: 97. doi:10.1186/s12915-014-0097-x

12. Oostra V, Brakefield PM, Hiltemann Y, Zwaan BJ, Brattström O. On the fate of seasonally plastic traits in a rainforest butterfly under relaxed selection. Ecol Evol. 2014;4: 2654–2667. doi:10.1002/ece3.1114

13. van Bergen E, Osbaldeston D, Kodandaramaiah U, Brattström O, Aduse-Poku K, Brakefield PM. Conserved patterns of integrated developmental plasticity in a group of polyphenic tropical butterflies. BMC Evol Biol. 2017;17: 59. doi:10.1186/s12862-017-0907-1
