# Supplementary material for: Polyphenism of visual and chemical secondary sexually-selected wing traits in the butterfly Bicyclus anynana: How different is the intermediate phenotype?
Source: PLoS One. 2019 Nov 18;14(11):e0225003. doi: 10.1371/journal.pone.0225003 (PMC6860419; doi:10.1371/journal.pone.0225003)
Supplement: S2 Table — (DOCX) [file pone.0225003.s004.docx]

# Supplementary material:

## Supplementary S2 Table

S2 Table. **Summaries of models testing for the changes in wing morphological traits, in secondary sexually-selected traits, and in secondary sexually-selected traits as residuals on polyphenic wing morphological traits across the four developmental temperatures (17°C, 21°C, 23°C, 27°C) in experiment A** (see methods).

| Trait type | Trait | Model terms | Estimate±1 SE | *t* value | *P* | Adjusted R² |
| --- | --- | --- | --- | --- | --- | --- |
| Morphological | hv5 area/Wing size²^a^ | intercept | -7.65e-2 ±4.32e-2 | -1.77 | 0.078 | 0.80 |
|  |  | dev. temperature | 13.17± 0.44 | 29.76 | **< 0.001** |  |
|  |  | dev. temperature² | -0.79± 0.44 | -1.80 | 0.074 |  |
|  |  | sex male | 0.15 ± 5.98e-2 | 2.45 | **0.015** |  |
|  |  | *dev. temperature *sex* | *-0.37 ± 0.88* | *-0.42* | *0.68* |  |
|  |  | *dev. temperature² *sex* | *-1.64 ± 0.88* | *-1.86* | *0.06* |  |
|  | hv2 area/Wing size²^a^ | intercept | -0.26±4.47e-2 | -5.89 | **< 0.001** | 0.79 |
|  |  | dev. temperature | 0.84±3.10e-2 | 27.14 | **< 0.001** |  |
|  |  | sex male | 0.50±6.18e-2 | 8.13 | **< 0.001** |  |
|  |  | dev. temperature *sex | *-5.53e-2 ±6.20e-2* | *-0.89* | *0.37* |  |
|  | fv2 area/Wing size²^a^ | intercept | -0.16 ±4.91e-2 | -3.24 | **0.001** | 0.75 |
|  |  | dev. temperature | 0.84±3.40e-2 | 24.79 | **< 0.001** |  |
|  |  | sex male | 0.30± 6.80e-2 | 4.48 | **< 0.001** |  |
|  |  | *dev. temperature *sex* | *6.20e-2±6.81e-2* | *0.91* | *0.36* |  |
|  | jct length/Wing size | intercept | 6.78e-2 ±2.25e-3 | 30.17 | **< 0.001** | 0.15 |
|  |  | dev. temperature | -0.19± 3.28e-2 | -5.69 | **< 0.001** |  |
|  |  | dev. Temperature² | -5.99e-2± 3.34e-2 | -1.80 | 0.07 |  |
|  |  | sex male | -3.18e-3 ± 3.11e-3 | -1.02 | 0.31 |  |
|  |  | dev. temperature *sex | 0.10 ±4.61e-2 | 2.25 | **0.03** |  |
|  |  | dev. temperature² *sex | 1.92e-2± 4.61e-2 | 0.42 | 0.68 |  |
| Sexually-selected traits | fd5 area/Wing size²^a^ | intercept | -1.45±8.89e-4 | -1627.92 | **<0.001** | 0.54 |
|  |  | dev. Temperature | 3.15e-3±6.17e-4 | 5.11 | **<0.001** |  |
|  |  | sex male | -2.02e-2±1.27e-3 | -15.88 | **<0.001** |  |
|  |  | *dev. temperature *sex* | *-3.06e-6±1.24e-3* | *-0.002* | *1.00* |  |
|  | fd5 relative  brightness^a^ | intercept | 6891.0±173.3 | 39.77 | **< 0.001** | 0.23 |
|  |  | dev. temperature | 213.2±171.5 | 1.24 | 0.22 |  |
|  |  | sex male | -1917.3± 247.3 | -7.75 | **< 0.001** |  |
|  |  | dev. temperature *sex | -947.9 ± 242.0 | -3.92 | **< 0.001** |  |
|  | MSP1 | intercept | 3673.2 ±123.6 | 29.72 | **< 0.001** | 0.14 |
|  |  | dev. temperature | 565.6±120.6 | 4.69 | **< 0.001** |  |
|  | MSP2 | intercept | 1184.02±36.90 | 32.09 | **< 0.001** | -0.01 |
|  |  | dev. temperature | 1.80±36.02 | 0.05 | 0.96 |  |
|  | MSP3 | intercept | 14797.8±501.5 | 29.51 | **< 0.001** | 0.16 |
|  |  | dev. temperature | 2532.0±490.9 | 5.16 | **< 0.001** |  |
|  | MSP2/MSP1^a^ | intercept | -1.08±4.07e-2 | -26.63 | **< 0.001** | 0.13 |
|  |  | dev. temperature | -2.82±0.67 | -4.20 | **< 0.001** |  |
|  |  | dev. Temperature² | -1.20±0.69 | -1.74 | 0.08 |  |
|  | MSP2/MSP3^a^ | intercept | -2.93±5.82e-2 | -50.31 | **< 0.001** | 0.14 |
|  |  | dev. temperature | -4.20±0.96 | -4.37 | **< 0.001** |  |
|  |  | dev. Temperature² | 1.85±0.99 | 1.88 | 0.06 |  |
|  | MSP1/MSP3 | intercept | 0.26±5.44e-3 | 47.15 | **< 0.001** | -0.006 |
|  |  | dev. temperature | -0.002±5.33e-3 | -0.44 | 0.66 |  |
| Sexually-selected trait residuals | fd5 area/Wing size^a^ | intercept | 1.30e-2 ±1.20e-3 | 10.84 | **0.001** | 0.53 |
|  |  | dev. temperature | 1.57e-3±8.48e-4 | 1.85 | 0.07 |  |
|  |  | sex male | -0.03± 1.69e-3 | -15.28 | **< 0.001** |  |
|  |  | *dev. temperature *sex* | *-5.93e-4± 1.70e-3* | *-0.35* | *0.73* |  |
|  | fd5 relative  brightness^a^ | intercept | 474.6±108.3 | 4.38 | **< 0.001** | 0.19 |
|  |  | dev. Temperature | 3971.4±1574.6 | 2.52 | **0.01** |  |
|  |  | dev. Temperature² | -3172.4±1561.4 | -2.03 | **0.04** |  |
|  |  | sex male | -922.8±152.8 | -6.04 | **< 0.001** |  |
|  |  | dev. temperature *sex | -7777.1 ± 2202.6 | -3.53 | **< 0.001** |  |
|  |  | dev. temperature² *sex | 2729.6 ± 2200.1 | 1.24 | 0.22 |  |
|  | MSP1 | intercept | 1.29e-13 ±1.43e2 | 0.00 | 1.00 | -0.01 |
|  |  | dev. temperature | 37.5±1.44e2 | 0.26 | 0.80 |  |
|  | MSP2 | intercept | -2.15e-14±41.7 | 0.00 | 1.00 | 0.01 |
|  |  | dev. temperature | -1.87e1±4.17e2 | -0.05 | 0.96 |  |
|  |  | dev. Temperature² | 7.51e2±4.17e2 | 1.80 | 0.08 |  |
|  | MSP3 | intercept | -9.17e-13±5.65e2 | 0.00 | 1.00 | -0.01 |
|  |  | dev. temperature | 39.2±5.68e2 | 0.07 | 0.95 |  |
|  | MSP2/MSP1^a^ | intercept | -7.49e-17±6.13e-2 | 0.00 | 1.00 | 0.02 |
|  |  | dev. temperature | 1.42e-2±0.61 | 0.02 | 0.98 |  |
|  |  | dev. Temperature² | 1.20±0.61 | 1.97 | 0.052 |  |
|  | MSP2/MSP3^a^ | intercept | -5.79e-17±0.12 | 0.00 | 1.00 | 0.04 |
|  |  | dev. temperature | 0.20±1.22 | 0.17 | 0.87 |  |
|  |  | dev. temperature² | 2.91±1.22 | 2.39 | **0.02** |  |
|  | MSP1/MSP3 | intercept | -1.09e-19±6.65e-3 | 0.00 | 1.00 | -0.003 |
|  |  | dev. temperature | 5.62e-3±6.69e-3 | 0.84 | 0.40 |  |

MSP: Male Sex Pheromone. fd5: posterior eyespot on dorsal forewing. Text in bold: *P*<0.05. ^a^Box-Cox transformed response variables. Rejected interaction terms (from the full model) are in italics, significant ones in bold. The intercept is for the minimal model including at least all fixed factors without interactions. ‘Dev.temperature²’ is the quadratic term of the linear term ‘Dev.temperature’ using the [R function poly(x,2)]. In order to standardize units of morphological wing trait ratios, forewing and hindwing interpupil distances (i.e. proxies of wing size) were squared when used to correct eyespot areas of hv5, hv2, fv2, fd5.
