# Supplementary material for: Polyphenism of visual and chemical secondary sexually-selected wing traits in the butterfly Bicyclus anynana: How different is the intermediate phenotype?
Source: PLoS One. 2019 Nov 18;14(11):e0225003. doi: 10.1371/journal.pone.0225003 (PMC6860419; doi:10.1371/journal.pone.0225003)
Supplement: S3 Table — (DOCX) [file pone.0225003.s005.docx]

# Supplementary material:

## Supplementary S3 Table

S3 Table. **Summaries of models testing for the changes in chemical secondary sexually-selected traits throughout male adult life, i.e. from 3 to 28 days at the rearing temperature of 20°C, and from 3 to 21 days at 27°C, in experiment B**.

| Trait | Model terms | Estimate±1 SE | *t* value | *P* | Adjusted R² |
| --- | --- | --- | --- | --- | --- |
| MSP1 | intercept | 1560.47 ± 97.79 | 15.96 | **< 0.001** | 0.48 |
|  | temperature | -506.62± 97.53 | -5.19 | **< 0.001** |  |
|  | age | -287.95 ± 1068.88 | -0.27 | 0.79 |  |
|  | age² | -4934.38 ± 1146.89 | -4.30 | **< 0.001** |  |
|  | temperature*age | -6090.68 ± 1027.09 | -5.93 | **< 0.001** |  |
|  | temperature*age² | 1762.10 ± 1099.28 | 1.60 | 0.11 |  |
| MSP2 | intercept | 44.11 ± 1.99 | 22.16 | **< 0.001** | 0.36 |
|  | temperature | 2.23 ± 2.12 | 1.05 | 0.30 |  |
|  | age | 111.81 ± 17.73 | 6.31 | **< 0.001** |  |
|  | age² | -36.40 ± 18.70 | -1.95 | 0.06 |  |
|  | temperature*age | *-35.51 ± 22.69* | *-1.57* | *0.12* |  |
|  | temperature*age² | *25.91 ± 24.28* | *1.01* | *0.29* |  |
| MSP3 | intercept | 30.54 ± 0.73 | 41.74 | **< 0.001** | 0.58 |
|  | temperature | -4.63 ± 0.73 | -6.34 | **< 0.001** |  |
|  | age | -16.20 ± 8.00 | -2.03 | **0.047** |  |
|  | age² | -41.95 ± 8.58 | -4.89 | **< 0.001** |  |
|  | temperature*age | -61.09 ± 7.69 | -7.95 | **< 0.001** |  |
|  | temperature*age² | 9.97 ± 8.23 | 1.21 | 0.23 |  |
| MSP2/MSP1 | intercept | 0.25 ± 0.02 | 12.57 | **< 0.001** | 0.35 |
|  | temperature | 0.08 ± 0.02 | 4.13 | **< 0.001** |  |
|  | age | 0.12 ± 0.02 | 5.54 | **< 0.001** |  |
|  | temperature*age | 0.05 ± 0.02 | 2.28 | **0.03** |  |
| MSP2/MSP3 | intercept | 0.05 ± 0.003 | 16.72 | **< 0.001** | 0.53 |
|  | Temperature | 0.02 ± 0.003 | 6.11 | **< 0.001** |  |
|  | Age | 0.03 ± 0.003 | 7.38 | **< 0.001** |  |
|  | temperature*age | 0.01 ± 0.003 | 4.39 | **< 0.001** |  |
| MSP1/MSP3 | intercept | -1.47 ± 0.05 | -28.05 | **< 0.001** | 0.41 |
|  | temperature | 0.21 ± 0.05 | 4.08 | **< 0.001** |  |
|  | Age | 1.66 ± 0.57 | 2.94 | **0.005** |  |
|  | age² | 1.29 ± 0.59 | 2.18 | **0.03** |  |
|  | temperature*age | 3.16 ± 0.55 | 5.71 | **< 0.001** |  |
|  | temperature*age² | -0.18± 0.58 | -0.31 | 0.76 |  |

MSP: Male Sex Pheromone. Text in bold: *P*<0.05. All response variables were Box-Cox transformed except MSP1. Rejected interaction terms (from the full model) are in italics, significant ones in bold. The intercept is for the minimal model including at least all fixed factors without interactions. ’age²’ is the quadratic term of the linear term ‘age’ using [R function poly(x,2)].
